# Role of human papillomavirus infection in the etiology of vulvar cancer in Italian women

**DOI:** 10.1186/s13027-020-00286-8

**Published:** 2020-04-01

**Authors:** Mario Preti, John Charles Rotondo, Dana Holzinger, Leonardo Micheletti, Niccolò Gallio, Sandrine McKay-Chopin, Christine Carreira, Sebastiana Silvana Privitera, Reiko Watanabe, Ruediger Ridder, Michael Pawlita, Chiara Benedetto, Massimo Tommasino, Tarik Gheit

**Affiliations:** 1 Department of Surgical Sciences, University of Turin, Turin, Italy; 2 International Agency for Research on Cancer, Lyon, France; 3 Department of Morphology, Surgery and Experimental Medicine; Section of Pathology, Oncology and Experimental Biology; Laboratories of Cell Biology and Molecular Genetics, University of Ferrara, Ferrara, Italy; 4 Infections and Cancer Epidemiology, Infections and Cancer Program, German Cancer Research Center (DKFZ), Heidelberg, Germany; 5 Department of Oncology, Città della Salute e della Scienza, University of Turin, Turin, Italy; 6 Roche mtm laboratories, Mannheim, Germany; 7 Ventana Medical Systems Inc., Tucson, AZ USA

**Keywords:** Cancer, Human papillomavirus, Lymph node metastatic tissues, Vulvar squamous cell carcinoma, Multiple markers of viral infections

## Abstract

**Background:**

Vulvar squamous cell carcinoma (VSCC) is a rare malignancy of the female genital tract. We aimed to determine the mucosal high-risk human papillomavirus (HPV)-attributable fraction of VSCCs from Italian women using multiple markers of viral infections.

**Methods:**

VSCCs and 8 metastatic lymph node samples from 107 Italian women were analyzed by a highly type-specific multiplex genotyping assay for the presence of DNA from 119 different HPVs. Tissues were further analyzed for HPV RNA and for upregulation of the cellular protein p16^INK4a^.

**Results:**

The rate of mucosal HPV-related tumors defined by viral DNA and RNA positivity was low (7.8%). HPV16 was the most prevalent, followed by 53, 56, and 58. Only five (4.9%) p16^INK4a^-positive tumors were also positive for both viral DNA and RNA. One (14.3%) metastatic lymph node sample was positive for all three markers. DNA of cutaneous HPVs was detected in only two VSCCs, i.e. genus beta types 5 and 110.

**Conclusion:**

A small proportion of Italian VSCCs is putatively HPV-related, i.e. positive for both viral DNA and RNA of the same type, thus reinforcing the importance of HPV vaccination. Moreover, this study suggests that a direct role of HPV from genus beta and gamma in vulvar carcinogenesis is unlikely.

## Introduction

Vulvar squamous cell carcinoma (VSCC) is a rare tumor of the female genital tract, accounting for about 5% of all gynecological malignancies [1, 2]. In North America, the incidence of VSCC has increased by 0.6% annually in the past decade [1, 3, 4]. Moreover, tumor nodal recurrence is typically fatal [1]. The primary treatment approach for VSCC is surgery, complemented in selected cases by chemotherapy/radiotherapy [5], with negative psychosexual consequences [1, 6]. VSCC includes two distinct types, with apparently different etiology: (i) VSCC associated with infection with mucosal high-risk (HR) human papillomaviruses (HPVs), and (ii) HPV-independent VSCC [7, 8]. HPV-related VSCC is more common in younger women and is always preceded by a pre-malignant lesion, i.e. vulvar high-grade squamous intraepithelial lesion (VHSIL) [9, 10]. HPV-independent VSCC occurs mainly in older women [1, 11] and arises from a chronic inflammatory dermatosis, referred to as lichen sclerosus [12, 13], that can evolve into differentiated vulvar intraepithelial neoplasia (VIN), a precursor lesion of VSCC [14].

Based on a pooled prevalence rate of HPV DNA, 34% of vulvar cancers have been reported to be attributable to HPV [15].

Although detection of HR-HPV DNA is a valid testing approach for the identification of pre-malignant and malignant cervical lesions, its use for other anatomical sites, including the vulva, is insufficient proof for viral causality and can lead to misclassification of the lesion [16, 17]. Therefore, the use of additional markers for active HR-HPV infections, such as overexpression of the human cyclin-dependent kinase-4 inhibitor p16^INK4a^ (p16) or/and detection of viral RNA, may enable more precise estimation of the proportion of VSCC that may be attributable to HPV, as previously shown in head and neck cancer [16].

As recently suggested, a useful protocol for the identification of HPV-related VSCC is the simultaneous detection of HPV DNA and RNA [16, 17].

The evaluation of p16 protein expression by immunohistochemistry (IHC) enables discrimination between HPV-independent and HPV-related cancers at anatomical sites outside the cervix, including the vulva [17–21]. However, because there are a limited number of studies evaluating p16 expression in VSCC, the actual reliability of p16 protein expression as a single marker of oncogenic HPV infection for this tumor entity is not entirely known [17, 18, 22, 23]. Detection of both HPV DNA and p16 overexpression to classify the two different VSCC subtypes has also been proposed, but for VSCC this algorithm has not yet been fully validated [17, 18, 21]. However, the detection of viral transcripts as a marker of biological HPV activity in the lesion tissue, in addition to HPV DNA and p16 overexpression, may significantly improve the classification of truly HPV-positive VSCC.

A multicenter study using HPV DNA and p16 positivity as markers has shown that approximately 25% of vulvar cancers worldwide may be associated with HPV [18]. This study, which included tumor specimens collected from cancer cases in 39 countries on five continents, highlighted some variability in HPV positivity in VSCC in the different geographical regions. However, some geographical variations in HPV-positive VSCC may be due simply to the analysis of a relatively low number of specimens in specific countries. Thus, additional studies are required in individual countries, where only limited information on the association between HR-HPV and VSCC is available.

Regarding HR-HPV-negative VSCC, the involvement of other infectious agents, including the cutaneous genus beta and gamma HPV types, has been poorly investigated. Studies have reported the presence of these HPV types in the anogenital tract [24, 25]. In addition, several investigations have highlighted the transforming properties of genus beta and gamma HPV types in various experimental models [26–28].

In this study, we aimed to determine the HPV-attributable proportion of VSCC by analyzing HPV DNA and RNA status, and by determining p16 expression level, in a large retrospective cohort of VSCC cases from Italy. In addition, we examined the possible role of genus beta and/or gamma HPV types in VSCC.

## Materials and methods

### Sample collection

The Ethics Committee of the Hospital of Turin approved this study to be in compliance with the Declaration of Helsinki (reference number 1005). All patients had signed informed consent at the time of hospital admission. The inclusion criteria were: invasive vulvar carcinoma (i.e. an invasive depth > 1 mm), squamous cell histological type, and no prior surgical treatment for vulvar cancer or any other malignancy (apart from biopsy). The exclusion criteria were: primary site other than vulva, recurrent tumors, HIV- or hepatitis C virus (HCV)-positive patients, and non-consenting patients.

Formalin-fixed, paraffin-embedded (FFPE) VSCC samples obtained from 107 women (mean [±standard deviation (SD)] age, 73 [11.8] years) collected during 2013–2016 were retrieved from the archive of the Department of Obstetrics and Gynecology, University of Turin, Sant’Anna Hospital, Turin, Italy. According to the International Federation of Gynecology and Obstetrics (FIGO) classification of vulvar cancer [29], the VSCCs were in the following stages: IB (54.2%); II (0.9%); IIIA (19.6%); IIIB (11.2%); IIIC (4.7%); and IVA (9.3%). The mean tumor diameter was 29 mm (SD, 15.8 mm), and 42.1, 45.8, and 12.1% of tumors showed depth of invasion of < 5 mm, 5–12 mm, and > 12 mm, respectively. Archived FFPE metastatic lymph node samples (MTS) were also available from 8 women with VSCC tissue (mean [±SD] age, 69 [16.5] years).

### Preparation of paraffin sections and tumor evaluation

Ten sections of 10 μm from each FFPE tissue block were processed at the Department of Pathology, University of Turin, Italy. Sections S1 and S10 were used for hematoxylin and eosin histology, and S2 and S9 were used for p16 IHC. In addition, S3–S5 and S6–S8 were collected for DNA and RNA extraction, respectively. To avoid cross-contamination between different samples, each time a new case was sectioned, the microtome was extensively washed with DNA Away (Dutscher, Brumath, France) and a new microtome blade was used. Empty paraffin blocks were cut after every 10 cancer specimens and blindly analyzed. Tumor size/status was documented by routine pathologic evaluation, in accordance with the World Health Organization (WHO) classification [30].

### Nucleic acid extraction

Total DNA was prepared by incubating overnight at 37 °C three internal FFPE sections (S3–S5) from each VSCC/MTS tissue in 250 μl of digestion buffer (10 mM Tris/HCl at pH 7.4, 0.5 mg/ml proteinase K, and 0.4% Tween 20). Then, to inactivate the proteinase K and to separate paraffin from the aqueous phase, samples were incubated at 95 °C for 10 min, centrifuged, and chilled on ice [31]. The aqueous phase was transferred to a new tube.

Total RNA was prepared from tissue sections S6–S8 using the PureLink FFPE Total RNA Isolation Kit (Invitrogen, Carlsbad, CA, USA). DNase I (RNase-Free DNase Set, Qiagen, Hilden, Germany) treatment was carried out on the RNA purifying columns during sample processing as previously described [32]. Extracted RNA was eluted in 50 μl of RNase-free water and stored at − 80 °C until use.

### HPV DNA detection and genotyping

HPV DNA was detected by E7 type-specific multiplex genotyping (E7-MPG), which combines multiplex polymerase chain reaction (PCR) and hybridization to type-specific oligonucleotide probes on fluorescent beads (Luminex Corp., Austin, TX, USA) [33, 34]. E7-MPG uses HPV type-specific primers targeting the E7 region of 12 HR-HPVs (HPV16, 18, 31, 33, 35, 39, 45, 51, 52, 56, 58, 59), 7 possible/probable HR (pHR)-HPVs (HPV26, 53, 66, 68, 70, 73, 82), and two low-risk (LR) HPVs (HPV6 and 11). Forty-six genus beta HPVs (HPV5, 8, 9, 12, 14, 15, 17, 19, 20, 21, 22, 23, 24, 25, 36, 37, 38, 47, 49, 75, 76, 80, 92, 93, 96, 98, 99, 100, 104, 105, 107, 110, 111, 113, 115, 118, 120, 122, 124, 143, 145, 150, 151, 152, 159, 174) and 52 genus gamma HPVs (HPV4, 48, 50, 60, 65, 88, 95, 101, 103, 108, 109, 112, 116, 119, 121, 123, 126, 127, 128, 129, 130, 131, 132, 133, 134, 148, 149, 156, 161, 162, 163, 164, 165, 166, 167, 168, 169, 170, 171, 172, 173, 175, 178, 179, 180, 184, 197, 199, 200, 201, 202, SD2) [35–41] were detected using the same methodology.

The sensitivity has been evaluated using serial dilutions of DNA from HPV types. This multiplex PCR protocol is highly sensitive, with the ability to detect only 10 copies of the viral genome [34, 40].

### HPV mRNA analysis

All HPV DNA-positive VSCC/MTS tissues, including HPV16 and non-HPV16 DNA-positive samples, and a group of 10 randomly selected HPV DNA-negative tissues were analyzed for HPV16 E6*I mRNA and, as a control for tissue and RNA quality, for human UbC mRNA. Tissues that were DNA-positive for HR- and pHR-HPV types other than HPV16 were also analyzed for E6*I mRNA of the specific HR- and pHR-HPV types(s) detected. In addition, tissues that were DNA-positive for LR-HPV6 or 11 were analyzed for the presence of unspliced E6 mRNA of HPV6 and 11, respectively. RNA detection from FFPE sections was performed as previously described [32]. Briefly, 1 μl of extracted RNA was subjected to a one-step reverse transcription PCR protocol with the QuantiTect Virus Kit (Qiagen, Hilden, Germany) using HPV type-specific primers to amplify 65–75 bp cDNA sequences across the E6*I splice sites [32]. The biotinylated strands of the amplicons were detected by hybridization with type- and splice site-specific oligonucleotide probes coupled to fluorescence-labelled Luminex beads (Luminex Corp., Austin, TX, USA). The E6*I mRNA assays are available for 20 HR- or pHR-HPV types for which existence of splice sites was demonstrated [32]. For detection of unspliced LR-HPV6 and 11 mRNA, primers designed to amplify a 77 bp amplicon of the full-length HPV6 or 11 E6 gene, and an oligonucleotide probe for detection of full-length HPV11 E6, were applied.

Samples that were negative in duplicate assays for HPV E6*I or E6 and for UbC mRNA were considered RNA-invalid.

### p16 immunohistochemistry

Expression of p16 was evaluated manually by IHC in FFPE sections using the CINtec p16 Histology Kit (Roche mtm laboratories AG, Mannheim, Germany) according to the manufacturer’s instructions and as previously described [42]. Expression of p16 was evaluated by IHC in all VSCC and MTS cases. Continuous, diffuse nuclear and cytoplasmic staining in 70% or more of the tumor cells was considered p16-positive, and focal staining or no staining was considered p16-negative. All slides were evaluated three times by three different evaluators (SSP, RW, RR), who were blinded to the clinical and epidemiological data. The final classification of the staining was based on the majority consensus.

## Results

### HPV infection markers in VSCCs

Of 107 VSCC and 8 MTS cases initially selected, five VSCC cases and one MTS case were excluded due to invalid RNA (*n* = 3) and p16 (*n* = 2) data, or to the absence of cancer tissue in the block (*n =* 1). Thus, a total of 102 VSCC and 7 MTS tissues were included in the analysis. The 102 VSCC cases had a mean age of 70 years (SD, 12.8 years); FIGO stages were IB (52.9%), II (1%), IIIA (20.6%), IIIB (10.8%), IIIC (4.9%), and IVA (9.8%). Mean tumor diameter was 30 mm (SD, 15.7 mm) and mean depth of stromal invasion 8 mm (SD, 6.3 mm), with 40.2, 47.1 and 12.7% of tumors showing depth of invasion of < 5 mm, 5–12 mm, and > 12 mm, respectively. Furthermore, MTS selected from 7 cases were analyzed (mean [±SD] age, 73.9 [13.5] years).

Table 1 and Supplementary Table S1 show the HPV DNA, RNA, and p16 detection in VSCC and MTS cases. Mucosal HPV DNA was detected in 17 of 102 (16.7%) VSCCs and all 7 MTS cases (100%). HPV16 was the most prevalent type; it was present in 10 of the 17 HPV DNA-positive VSCCs (58.8%), followed by HPV6 (2/17, 11.8%), HPV18 (1/17, 5.9%), HPV53 (1/17, 5.9%), HPV56 (1/17, 5.9%), HPV58 (1/17, 5.9%), and HPV82 (1/17, 5.9%). Only one multiple infection was detected, containing HPV16, 18, and 56. All MTS cases were positive for mucosal HPV DNA. Five MTS cases contained a single HPV type, i.e. HPV16 (*n* = 1), HPV6 (*n* = 3), and HPV56 (*n* = 1). Multiple HPV infections were detected in two MTS cases; both were positive for HPV6 and HPV16 (Table 1). Matched VSCC and MTS cases of three women were positive for the same HPV DNA, i.e. HPV6 (*n* = 2) and HPV16 (*n* = 1). There was no concordance for the HPV types in the remaining MTS cases (*n* = 4) and the corresponding VSCCs (Supplementary Table S1).

**Table 1:** HPV DNA, RNA, and p16 positivity in 102 VSCC and 7 MTS cases

		VSCC (*N* = 102)	MTS (*N* = 7)
**HPV type**	**Marker positivity**	Positive *n* (%)	Positive *n* (%)
**Any HPV**	DNA	17 (16.7)	7 (100.0)
	DNA and RNA^a^	8 (7.8)	1 (14.3)
	DNA, RNA, and p16^a^	5 (4.9)	1 (14.3)
**HPV16 single infection**	DNA	9 (8.8)	1 (14.3)
	DNA and RNA	5 (4.9)	0 (0.0)
	DNA, RNA, and p16	4 (3.9)	0 (0.0)
**Mucosal HPV other than HPV16 single infections**	DNA	7 (6.9)^b^	4 (57.1)^d^
	DNA and RNA	2 (2.0)^c^	0 (0.0)
	DNA, RNA, and p16	0 (0.0)	0 (0.0)
**Multiple infections**	DNA	1 (1.0)^e^	2 (28.6)^g^
	DNA and RNA	1 (1.0)^f^	1 (14.3)^h^
	DNA, RNA, and p16	1 (1.0)	1 (14.3)

*HPV* Human papillomavirus, *MTS* Metastatic lymph node samples, *VSCC* Vulvar squamous cell carcinoma

^a^HPV RNA was examined in 17 VSCC and 7 MTS HPV DNA-positive cases and a randomly selected subset of HPV DNA-negative cases (*n* = 10). p16 expression was examined in all VSCC (*n* = 102) and MTS (*n* = 7) cases

^b^Single infections: HPV6 (*n* = 2), HPV18 (*n* = 1), HPV53 (*n* = 1), HPV56 (*n* = 1), HPV58 (*n* = 1), HPV82 (*n* = 1)

^c^Single infections: HPV53 (*n* = 1), HPV58 (*n* = 1)

^d^Single infections: HPV6 (*n* = 3), HPV56 (*n* = 1)

^e^Coinfection: HPV16, HPV18, and HPV56 (*n* = 1)

^f^Coinfection: HPV16 and HPV56 (*n* = 1)

^g^Coinfection: HPV6 and HPV16 (*n* = 2)

^h^Single infection: HPV16 (*n* = 1)

To evaluate the etiological role of the mucosal HPV detected, all 17 HPV DNA-positive VSCC and all 7 MTS cases were analyzed for the presence of HR- and pHR-HPV E6*I and unspliced E6 for LR-HPV (Table 1). All 24 HPV DNA-positive VSCC and MTS cases were ubC mRNA-positive. Considering both HPV DNA and RNA positivity, the percentage of HPV-related VSCCs was 7.8% (8/102), i.e. HPV16 (*n* = 6), HPV53 (*n* = 1), HPV56 (*n* = 1), and HPV58 (*n* = 1). One VSCC case simultaneously tested positive for HPV DNA and RNA from HPV16 and HPV56. Of the 7 mucosal HPV DNA-positive MTS cases, only one (14.3%) had E6*I mRNA of the same type (HPV16). None of the HPV6 DNA-positive cases tested positive for full-length HPV6 E6 mRNA.

**Fig. 1:**
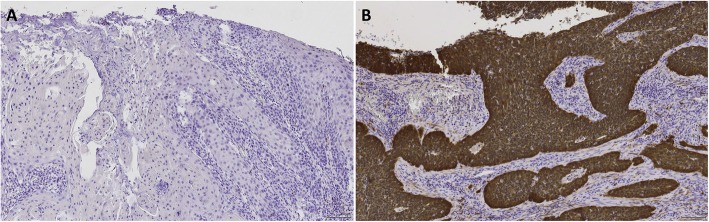
p16^ink4a^ immunohistochemistry staining. Negative p16^ink4a^ staining (**a**), and diffuse nuclear and cytoplasmic p16^ink4a^ staining (**b**) in VSCC cases. Magnification X10

Overexpression of the cell-cycle inhibitor p16 is a well-validated surrogate marker of HPV transformation in the cervix [43]. We determined the p16 IHC positivity in all 102 VSCC and 7 MTS cases (Fig. 1). Eleven VSCC cases were positive for p16 (11/102, 10.8%). Of these, five were also HPV DNA- and RNA-positive (Table 1). Thus, when all three markers were considered, the percentage of HPV-related VSCC cases dropped from 7.8 to 4.9% (5/102) (Table 1). The remaining p16-positive cases (*n* = 6) were HPV DNA-positive and HPV RNA-negative (*n* = 2) or HPV DNA-negative and HPV RNA-negative (*n* = 2), or were not tested for HPV DNA and RNA (*n* = 2). Only one MTS case showed p16 overexpression (1/7, 14.3%), and was also positive for HPV16 DNA and RNA. The corresponding VSCC from the same case was also positive for all three markers.

The presence of DNA of cutaneous beta and gamma HPV in cytological samples of the genital tract has been reported [24, 37, 44]. We also determined the presence of DNA of 46 genus beta and 52 genus gamma HPV types in all VSCC and MTS cases. Only two VSCC cases were positive for beta HPV types (i.e. HPV5 and HPV110), and no gamma HPV types were detected in the VSCC and MTS cases analyzed.

## Discussion

Mucosal HR-HPV types have been clearly associated with the development of a small subset of VSCC. However, the contribution of the viral infectious agents appears to vary in different geographical regions [15]. In particular, limited information is available for the contribution of HR-HPV to VSCC development in Italy. In this study, we analyzed a large number of VSCCs collected in a hospital in northern Italy during 2013–2016.

In the first step, we investigated the presence of HPV DNA in VSCCs by using a sensitive HPV genotyping approach covering all oncogenic and probably/possibly oncogenic HPV types. The prevalence of HPV DNA-positive cases was observed to be nearly 17%. Previous studies on VSCC reported HPV DNA frequencies ranging from 29 to 45% [17, 18, 21, 45, 46]. However, the overall results indicate that HPV could have a role in the development of a proportion of VSCC cases. In agreement with a previous study [17], we observed HPV16 to be the most common HR-HPV type detected in VSCC. In addition, viral DNA from HPV18, 53, 56, 58, and 82 was also detected, with low frequency. The LR-HPV type 6 was found to be the second most common viral type in this study, suggesting that it may be involved, together with other factors, in VSCC onset or progression [17, 46]. Recently, Faber et al. (2017) reported a prevalence of 6.8% (19/277) for HPV6 DNA as a single infection in VIN, and 1.1% (6/527) in VSCC [47].

Because HPV transcriptional activity may reflect its oncogenic activity, we applied a HPV-type-specific E6*I mRNA detection method, established for 20 HR- or pHR-HPV types and validated for use in FFPE tissues, to evaluate the presence of HPV RNA in HPV DNA-positive VSCCs. We observed viral RNA in 47.1% (8/17) of HPV DNA-positive VSCC cases, with HPV16 RNA showing the highest prevalence. Eight of 102 VSCC cases (7.8%) were likely to be HPV-driven, i.e. positive for both HPV DNA and RNA. None of the HPV6 DNA-positive samples tested positive for HPV RNA. The upregulation of p16 has been suggested as a surrogate marker for oncogenic HR-HPV infections, and p16 IHC has frequently been used not only to determine and/or confirm positivity for HPV in various tumors but also to define HPV-related tumors [19]. Therefore, p16 expression has also been studied in VSCC before [17, 22, 48, 49]. In the present study, this cellular marker was overexpressed in approximately 50% of HR-HPV DNA-positive VSCC cases (7/15). Other series of VSCC studies previously showed 70–80% of HPV DNA-positive VSCC cases expressing p16 [17, 46]. However, in our study, p16 overexpression was observed in the majority (5/8, 62.5%) of HR-HPV DNA- and RNA-positive VSCCs. A lower p16 positivity rate in HR-HPV DNA-negative VSCCs (4/85, 4.7%) and in HR-HPV DNA-positive and HPV RNA-negative VSCCs (2/7, 28.6%) was also detected. Overexpression of p16 in HPV DNA-negative VSCCs may be a consequence of either a mutation of *CDKN2A*, the gene coding for p16 [50], or the inactivation of the retinoblastoma protein pRb (e.g. by mutation), which then leads to upregulation of p16 [51].

Previous reports showed higher concordance between the HR-HPV transcript expression and p16 overexpression, ranging from 83 to 90% [17, 21, 49]. The causes of this discrepancy between our results and those of previous published studies are unknown.

In this study, we were also able to retrieve MTSs for a small number of patients with VSCC. Only one case showed concordance in HPV DNA/RNA positivity and p16 expression between VSCC and MTS.

The investigation of the presence of HR-HPV E6 mRNA instead of analyzing E6 oncoprotein, which may be more informative on the oncogenic implications of HR-HPVs in VSCC development, may be considered a limitation of this study. However, we focused our attention on evaluating the expression of the p16 protein, which is considered a surrogate marker for all transforming HR-HPV infections and represents a single target molecule, different from the E6 proteins with type-specific polypeptide sequence variability.

Finally, although independent studies have reported that cutaneous beta and gamma HPV types can be detected in the genital tract, our study does not provide evidence for their involvement in the development of VSCC.

## Conclusion

We characterized the HPV-independent and HPV-related VSCCs in a cohort of more than 100 VSCC cases from Italy. We identified as putative HPV-related tumors a small proportion of VSCC cases that tested positive for both HPV DNA and RNA. Our results support the causative role of HPV in vulvar carcinogenesis in a distinct subset of cases and highlight the prevalence of HPV16, thus reinforcing the importance of HPV vaccination programs.

## Supplementary information

**Additional file 1: Table S1.** HPV DNA, HPV RNA and p16 status in 102 VSCC and 7 MTS individual cases.

## Data Availability

The dataset used for this study is available from the corresponding author.

## Abbrevations

VSCC: Vulvar squamous cell carcinoma
VHSIL: Vulvar high-grade squamous intraepithelial lesion
HPV: Human papillomavirus
HR: High-risk
FFPE: Formalin-fixed, paraffin-embedded
IHC: Immunohistochemistry
WHO: World Health Organization
