# Supplementary material for: Role of human papillomavirus infection in the etiology of vulvar cancer in Italian women
Source: Infect Agent Cancer. 2020 Apr 1;15:20. doi: 10.1186/s13027-020-00286-8 (PMC7110671; doi:10.1186/s13027-020-00286-8)
Supplement: Supplementary file 1 — Additional file 1: Table S1. HPV DNA, HPV RNA and p16 status in 102 VSCC and 7 MTS individual cases. [file 13027_2020_286_MOESM1_ESM.docx]

| **Supplementary Table 1:** HPV DNA, HPV RNA and p16 status in 102 VSCC and 7 MTS individual cases. | | | | | | | |
| --- | --- | --- | --- | --- | --- | --- | --- |
|  |  |  |  |  |  |  |  |
|  | **Vulvar squamous cell carcinoma** | | | **Corresponding lymph nodes metastasis** | | | |
| **ID numbers** | **HPV DNA** | **HPV RNA** | **p16** | **ID numbers** | **HPV DNA** | **HPV RNA** | **p16** |
| **TO 022** | **6** | **NEG** | **NEG** | **MTS-1** | **6, 16** | **NEG** | **NEG** |
| **TO 025** | **6** | **NEG** | **NEG** | **MTS-2** | **6** | **NEG** | **NEG** |
| **TO 076** | **53** | **53** | **NEG** | **MTS-4** | **16** | **NEG** | **NEG** |
| **TO 103** | **16** | **16** | **POS** | **MTS-5** | **6** | **NEG** | **NEG** |
| **TO 105** | **16** | **NEG** | **POS** | **MTS-7** | **56** | **NEG** | **NEG** |
| **TO 128** | **16** | **16** | **POS** | **MTS-3** | **6, 16** | **16** | **NEG** |
| **TO 135** | **16** | **16** | **POS** | **MTS-6** | **6** | **NEG** | **NEG** |
| **TO 014** | **82** | **NEG** | **NEG** |  |  |  |  |
| **TO 037** | **16** | **16** | **NEG** |  |  |  |  |
| **TO 040** | **18** | **NEG** | **POS** |  |  |  |  |
| **TO 044** | **16** | **NEG** | **NEG** |  |  |  |  |
| **TO 071** | **16** | **16** | **POS** |  |  |  |  |
| **TO 074** | **16** | **NEG** | **NEG** |  |  |  |  |
| **TO 102** | **56** | **NEG** | **NEG** |  |  |  |  |
| **TO 118** | **58** | **58** | **NEG** |  |  |  |  |
| **TO 120** | **16, 18, 56** | **16, 56** | **POS** |  |  |  |  |
| **TO 127** | **16** | **NEG** | **NEG** |  |  |  |  |
| **TO 004** | **NEG** | **NEG** | **NEG** |  |  |  |  |
| **TO 038** | **NEG** | **NEG** | **NEG** |  |  |  |  |
| **TO 053** | **NEG** | **NEG** | **NEG** |  |  |  |  |
| **TO 072** | **NEG** | **NEG** | **NEG** |  |  |  |  |
| **TO 082** | **NEG** | **NEG** | **NEG** |  |  |  |  |
| **TO 085** | **NEG** | **NEG** | **NEG** |  |  |  |  |
| **TO 110** | **NEG** | **NEG** | **NEG** |  |  |  |  |
| **TO 121** | **NEG** | **NEG** | **POS** |  |  |  |  |
| **TO 134** | **NEG** | **NEG** | **POS** |  |  |  |  |
| **TO 139** | **NEG** | **NEG** | **NEG** |  |  |  |  |
| **TO 002** | **NA*** | **NA** | **NEG** |  |  |  |  |
| **TO 005** | **NA** | **NA** | **NEG** |  |  |  |  |
| **TO 015** | **NA** | **NA** | **NEG** |  |  |  |  |
| **TO 016** | **NA** | **NA** | **NEG** |  |  |  |  |
| **TO 018** | **NA** | **NA** | **NEG** |  |  |  |  |
| **TO 019** | **NA** | **NA** | **NEG** |  |  |  |  |
| **TO 020** | **NA** | **NA** | **NEG** |  |  |  |  |
| **TO 021** | **NA** | **NA** | **NEG** |  |  |  |  |
| **TO 023** | **NA** | **NA** | **NEG** |  |  |  |  |
| **TO 024** | **NA** | **NA** | **NEG** |  |  |  |  |
| **TO 027** | **NA** | **NA** | **NEG** |  |  |  |  |
| **TO 028** | **NA** | **NA** | **NEG** |  |  |  |  |
| **TO 029** | **NA** | **NA** | **NEG** |  |  |  |  |
| **TO 030** | **NA** | **NA** | **NEG** |  |  |  |  |
| **TO 032** | **NA** | **NA** | **NEG** |  |  |  |  |
| **TO 033** | **NA** | **NA** | **NEG** |  |  |  |  |
| **TO 034** | **NA** | **NA** | **NEG** |  |  |  |  |
| **TO 035** | **NA** | **NA** | **NEG** |  |  |  |  |
| **TO 036** | **NA** | **NA** | **NEG** |  |  |  |  |
| **TO 039** | **NA** | **NA** | **NEG** |  |  |  |  |
| **TO 041** | **NA** | **NA** | **NEG** |  |  |  |  |
| **TO 042** | **NA** | **NA** | **NEG** |  |  |  |  |
| **TO 043** | **NA** | **NA** | **NEG** |  |  |  |  |
| **TO 045** | **NA** | **NA** | **NEG** |  |  |  |  |
| **TO 048** | **NA** | **NA** | **NEG** |  |  |  |  |
| **TO 049** | **NA** | **NA** | **NEG** |  |  |  |  |
| **TO 050** | **NA** | **NA** | **NEG** |  |  |  |  |
| **TO 051** | **NA** | **NA** | **NEG** |  |  |  |  |
| **TO 052** | **NA** | **NA** | **NEG** |  |  |  |  |
| **TO 054** | **NA** | **NA** | **NEG** |  |  |  |  |
| **TO 055** | **NA** | **NA** | **NEG** |  |  |  |  |
| **TO 056** | **NA** | **NA** | **NEG** |  |  |  |  |
| **TO 057** | **NA** | **NA** | **NEG** |  |  |  |  |
| **TO 059** | **NA** | **NA** | **NEG** |  |  |  |  |
| **TO 060** | **NA** | **NA** | **NEG** |  |  |  |  |
| **TO 061** | **NA** | **NA** | **NEG** |  |  |  |  |
| **TO 062** | **NA** | **NA** | **NEG** |  |  |  |  |
| **TO 064** | **NA** | **NA** | **NEG** |  |  |  |  |
| **TO 065** | **NA** | **NA** | **NEG** |  |  |  |  |
| **TO 066** | **NA** | **NA** | **NEG** |  |  |  |  |
| **TO 067** | **NA** | **NA** | **NEG** |  |  |  |  |
| **TO 068** | **NA** | **NA** | **NEG** |  |  |  |  |
| **TO 069** | **NA** | **NA** | **NEG** |  |  |  |  |
| **TO 070** | **NA** | **NA** | **NEG** |  |  |  |  |
| **TO 073** | **NA** | **NA** | **NEG** |  |  |  |  |
| **TO 075** | **NA** | **NA** | **NEG** |  |  |  |  |
| **TO 077** | **NA** | **NA** | **NEG** |  |  |  |  |
| **TO 078** | **NA** | **NA** | **NEG** |  |  |  |  |
| **TO 080** | **NA** | **NA** | **NEG** |  |  |  |  |
| **TO 081** | **NA** | **NA** | **NEG** |  |  |  |  |
| **TO 083** | **NA** | **NA** | **NEG** |  |  |  |  |
| **TO 084** | **NA** | **NA** | **NEG** |  |  |  |  |
| **TO 086** | **NA** | **NA** | **NEG** |  |  |  |  |
| **TO 087** | **NA** | **NA** | **NEG** |  |  |  |  |
| **TO 100** | **NA** | **NA** | **POS** |  |  |  |  |
| **TO 104** | **NA** | **NA** | **NEG** |  |  |  |  |
| **TO 106** | **NA** | **NA** | **NEG** |  |  |  |  |
| **TO 107** | **NA** | **NA** | **NEG** |  |  |  |  |
| **TO 112** | **NA** | **NA** | **NEG** |  |  |  |  |
| **TO 115** | **NA** | **NA** | **NEG** |  |  |  |  |
| **TO 116** | **NA** | **NA** | **NEG** |  |  |  |  |
| **TO 117** | **NA** | **NA** | **NEG** |  |  |  |  |
| **TO 119** | **NA** | **NA** | **NEG** |  |  |  |  |
| **TO 122** | **NA** | **NA** | **NEG** |  |  |  |  |
| **TO 123** | **NA** | **NA** | **NEG** |  |  |  |  |
| **TO 124** | **NA** | **NA** | **NEG** |  |  |  |  |
| **TO 125** | **NA** | **NA** | **POS** |  |  |  |  |
| **TO 126** | **NA** | **NA** | **NEG** |  |  |  |  |
| **TO 129** | **NA** | **NA** | **NEG** |  |  |  |  |
| **TO 130** | **NA** | **NA** | **NEG** |  |  |  |  |
| **TO 131** | **NA** | **NA** | **NEG** |  |  |  |  |
| **TO 132** | **NA** | **NA** | **NEG** |  |  |  |  |
| **TO 136** | **NA** | **NA** | **NEG** |  |  |  |  |
| **TO 137** | **NA** | **NA** | **NEG** |  |  |  |  |
| **TO 138** | **NA** | **NA** | **NEG** |  |  |  |  |
| *NA; Not available | |  |  |  |  |  |  |
